# Needle-Guided Scleral Fixation: A New Single-Suture Approach

**DOI:** 10.3390/jcm15010078

**Published:** 2025-12-22

**Authors:** Laura De Luca, Giovanni William Oliverio, Maura Mancini, Rino Frisina, Feliciana Menna, Stefano Lupo, Pierluigi Grenga, Cosimo Mazzotta, Pasquale Aragona, Alessandro Meduri

**Affiliations:** 1 Ophthalmology Clinic, Department of Biomedical and Dental Sciences and Morphofunctional Imaging, University of Messina, 98125 Messina, Italy; laura.deluca21@gmail.com (L.D.L.); gioliverio@unime.it (G.W.O.);; 2 Ophthalmology Unit, Surgery Department, Piacenza Hospital, 29121 Piacenza, Italy; frisinarino@gmail.com; 3 Department of Medical-Surgical Sciences and Biotechnologies, U.O.C. Ophthalmology, Sapienza University of Rome, Via Firenze 1, 04019 Terracina, Italy; 4 Ophthalmology Unit UOC, ASL Roma 3 G.B. Grassi Hospital, Ostia Lido, 00122 Rome, Italy; 5 Faculty of Medicine and Surgery, University of Enna “Kore”, 94100 Enna, Italy

**Keywords:** scleral fixation, aphakia, IOL malposition, needle-guided scleral fixation

## Abstract

**Background:** Scleral fixation of intraocular lenses (IOLs) is a valuable option in cases of aphakia or inadequate capsular support, yet conventional sutured and sutureless approaches can pose technical challenges and complication risks. The needle-guided scleral fixation technique offers a simplified, single-suture solution that enhances safety and reproducibility. **Methods:** In this retrospective interventional case series, 30 eyes with insufficient capsular support underwent IOL implantation using Meduri’s needle-guided single-suture technique at the G. Martino University Hospital, Messina. The surgical method employs a 24-gauge needle to guide a double-armed 10-0 polypropylene suture through the sclera for precise IOL anchorage, minimizing vitreous manipulation. Outcomes were assessed over 24 months, including best-corrected visual acuity (BCVA), IOL centration, intraocular pressure (IOP), and postoperative complications. **Results:** Mean BCVA improved from X to Y LogMAR at two years (*p* < 0.05). All IOLs remained well-centered without tilt or decentration. Mild conjunctival hyperemia occurred in 70% of cases, resolving spontaneously. No suture erosion, vitreous hemorrhage, or retinal detachment was observed. **Conclusions:** The needle-guided single-suture technique provides a stable, efficient, and reproducible method for posterior chamber IOL fixation in aphakic eyes lacking capsular support. Its minimal learning curve and reduced surgical complexity make it an attractive alternative to both traditional sutured and modern sutureless methods, particularly in centers without vitreoretinal expertise.

## 1. Introduction

Scleral fixation of intraocular lenses (IOLs) remains one of the most effective surgical options for visual rehabilitation in patients lacking adequate capsular support due to trauma, iatrogenic zonular rupture, or congenital anomalies [1,2,3]. In these cases, placement of an IOL in the posterior chamber is anatomically desirable, as it maintains a physiological lens position and minimizes corneal endothelial damage and iris trauma [4,5,6].

Historically, fixation techniques have evolved substantially. Early approaches, such as those developed by Malbran and Binkhorst in the 1970s, relied on suturing posterior chamber IOLs to the sclera using 10-0 polypropylene [7]. While effective, these procedures carried a risk of late suture breakage, lens tilt, and endophthalmitis. Over subsequent decades, advancements such as ab externo techniques, Hoffman pockets, and transconjunctival passages improved reproducibility and cosmetic outcomes [8,9].

In the past decade, sutureless techniques have gained popularity, with landmark contributions from Yamane’s double-needle flanged method [10,11], Agarwal’s glued IOL [12], and the Carlevale lens [13,14,15]. These have reduced suture-related complications but remain technically demanding, requiring a steep learning curve and specialized instruments. IOL tilt, haptic slippage, and postoperative hypotony remain concerns in less experienced hands [16,17,18].

To address these challenges, simplified approaches have been proposed. Meduri’s needle-guided single-suture technique, first reported as a conference abstract in 2024, represents the earliest published description of this simplified approach [19]. Although the first reference describing the technique is a conference abstract, it remains the earliest documentation of the surgical concept. No peer-reviewed reports predating this abstract were available. Meduri’s needle-guided single-suture scleral fixation offers an accessible and reproducible technique, suitable even for anterior segment surgeons without vitreoretinal expertise. By utilizing a 24-gauge needle as a guide for precise transscleral passage and a single-point anchorage, this method minimizes vitreous traction and avoids complex tunnel creation.

The present study reports clinical outcomes of this new single-suture technique with a 2-year follow-up, analyzing its safety, effectiveness, and reproducibility compared with other methods reported in the literature. Globally, the surgical management of aphakia without capsular support continues to evolve as the demographic of post-cataract patients expands. The World Health Organization estimates that nearly 10% of cataract surgeries worldwide require alternative fixation strategies due to capsular rupture or zonular dialysis. In developing countries, where access to vitreoretinal expertise is limited, simplified scleral fixation techniques that minimize equipment dependency are particularly valuable. Moreover, in teaching hospitals, the availability of a reproducible and safe technique such as the needle-guided single-suture approach offers a structured learning pathway for young surgeons, fostering skill acquisition without exposing patients to undue surgical risk. These educational and socioeconomic considerations underline the global relevance of this method as a bridge between complex modern procedures and the need for accessible, high-quality ophthalmic care.

## 2. Materials and Methods

**Figure 1:**
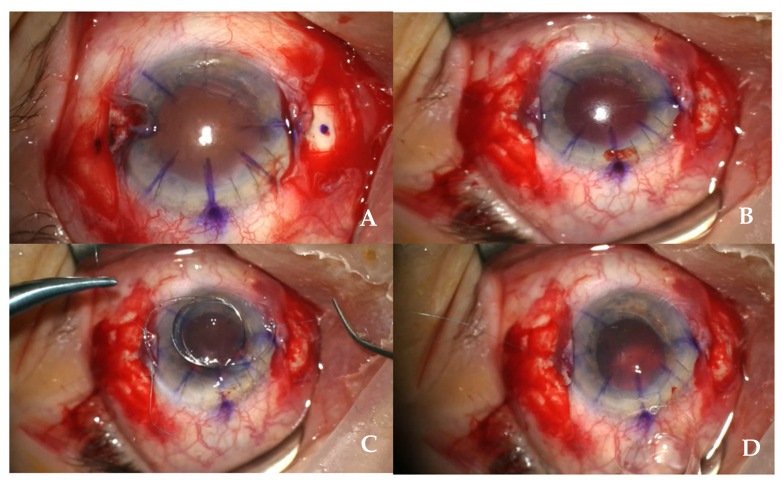
Surgical Technique: (**A**) Corneal Incision and Initial Steps: A 2.75 mm corneal incision was made for IOL insertion. Additional paracentesis was performed if fluid control was needed. Anterior vitrectomy was carried out in 10 cases where vitreous prolapse was present. Scleral Tunnel Creation: Corneal marking, conjunctival dissection, and two sclerotomies were performed using a 24-gauge needle, 3 mm posterior to the limbus at 3 and 9 o’clock. Sclerotomies were angled to avoid penetrating the vitreous cavity. (**B**) Needle-Guided Suture Passage: With the 24-gauge needle in place, a double-armed 10-0 polypropylene suture with one straight and one curved end was passed from the nasal to temporal scleral tunnel using the 24-gauge needle as a guide. The suture was then externalized through the corneal incision and cut in half. (**C**) Suture Fixation of IOL: The PMMA IOL was anchored to the suture ends through its preexisting holes. The IOL was then loaded into an injector and positioned through the corneal incision. Rotational forces applied to the suture ends ensured optimal centration. The suture was secured with a single knot on each side. (**D**) Wound Closure and Postoperative Care: The scleral tunnels and corneal incision were sealed, and conjunctival closure was performed with sutures.

This retrospective interventional case series included 30 eyes of 30 patients who underwent scleral fixation of an IOL using Meduri’s needle-guided single-suture technique between January 2022 and June 2023 at the Ophthalmology Clinic of the University of Messina, Italy. The study adhered to the tenets of the Declaration of Helsinki and received approval from the Institutional Review Board (Protocol No. 341/2022). Written informed consent was obtained from all participants. Inclusion criteria were aphakia or decentered IOL with insufficient capsular support, the absence of active ocular inflammation or infection, and a minimum postoperative follow-up of 24 months. Exclusion criteria included uncontrolled glaucoma, corneal opacities preventing adequate visualization, a history of retinal detachment or intraocular tumors, and severe systemic disease precluding surgery. Baseline evaluation included slit-lamp biomicroscopy, fundus examination, intraocular pressure measurement, and optical biometry using the IOLMaster 700 (Carl Zeiss Meditec, Jena, Germany). Corneal tomography (Pentacam, Oculus, Wetzlar, Germany) was performed to evaluate anterior segment parameters and scleral thickness. The IOL power was calculated using the SRK/T formula. All surgeries were performed by a single experienced surgeon (A.M.) under peribulbar anesthesia. Following aseptic preparation, a 2.75 mm clear corneal incision was made at the superior limbus. A 24-gauge needle was introduced 3 mm posterior to the limbus at 3 and 9 o’clock, creating transscleral tunnels directed tangentially to the globe to prevent vitreous entry. A double-armed 10-0 polypropylene suture was passed through the needle from nasal to temporal sides, externalized through the corneal incision, and then cut in half. The ends were tied to the eyelets of a single-piece PMMA IOL, which was then inserted and fixated by gentle traction, ensuring centration. Each suture end was secured with a single knot within the scleral tunnel, and the conjunctiva was closed with absorbable sutures. In cases with vitreous prolapse, a limited anterior vitrectomy was performed. The procedure required no vitrectomy ports or fibrin glue (Figure 1).

All patients received topical antibiotic–steroid combination drops four times daily for four weeks, followed by tapering anti-inflammatory drops. Follow-up visits were scheduled on postoperative days 1, 10, 30, 90, 180, and 720.

Primary outcomes included: BCVA (LogMAR) at 1, 6, 12, and 24 months; IOL centration and presence of tilt (evaluated by slit-lamp and anterior segment OCT); IOP changes; complication rates (suture erosion, endophthalmitis, retinal detachment).

Data were analyzed using SPSS v27.0 (IBM Corp., Armonk, NY, USA). Continuous variables were expressed as mean ± standard deviation (SD). Comparisons between preoperative and postoperative BCVA and IOP were performed using the Wilcoxon signed-rank test or paired *t*-test, as appropriate. Statistical significance was set at *p* < 0.05. Prior to surgery, all patients underwent a standardized preoperative work-up, including optical coherence tomography (OCT) to exclude macular pathology, endothelial cell count measurement, and corneal topography. Surgeries were performed under peribulbar anesthesia, with intravenous sedation (midazolam 0.05 mg/kg) when required for patient comfort. In cases of intraoperative miosis, intracameral adrenaline was administered to optimize visualization.

Postoperative care followed a uniform regimen: topical levofloxacin 0.5% and dexamethasone 0.1% four times daily for four weeks, tapered gradually, with artificial tears as needed. Follow-up visits occurred at days 1, 10, and 30, then at 3, 6, 12, and 24 months. At each visit, visual acuity, IOP, and anterior segment findings were documented, and any complications were recorded. Statistical analysis included verification of data normality using the Shapiro–Wilk test, and results were expressed as mean ± standard deviation (SD) with 95% confidence intervals (CIs). For continuous variables, the paired *t*-test or Wilcoxon signed-rank test was applied as appropriate, while categorical data were analyzed using Fisher’s exact test.

## 3. Results

A total of 30 eyes from 30 patients (18 males, 12 females) were included in the study. The mean age was 66.4 ± 9.7 years (range: 48–82 years). The primary indications for surgery were: dislocated intraocular lens (IOL): 12 eyes (40.0%), post-traumatic aphakia: 9 eyes (30.0%), zonular dialysis after complicated cataract surgery: 7 eyes (23.3%), congenital ectopia lentis: 2 eyes (6.7%). The mean follow-up period was 24.3 ± 2.1 months (range: 22–27 months). The mean preoperative best-corrected visual acuity (BCVA) was 0.89 ± 0.32 LogMAR (approx. Snellen equivalent 20/160) (Table 1).

**Figure 2:**
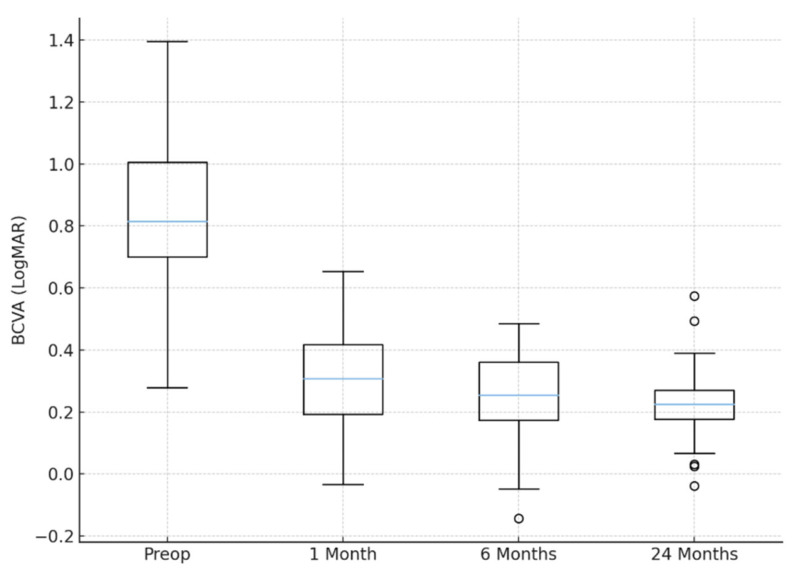
Boxplots illustrating best-corrected visual acuity (BCVA, LogMAR) at baseline and during postoperative follow-up (1, 6, and 24 months). Each box represents the interquartile range (IQR), with the horizontal line indicating the median value; whiskers extend to 1.5× IQR, and individual points denote outliers. The distribution shows a progressive and consistent shift toward lower (better) BCVA values over time, with reduced variability beginning from the 6-month visit onward.

Postoperatively, BCVA improved significantly to: 0.32 ± 0.18 LogMAR (20/40) at 1 month, 0.25 ± 0.15 LogMAR (20/35) at 6 months, and 0.23 ± 0.14 LogMAR (20/33) at 24 months (*p* < 0.001, paired *t*-test). A gain of ≥3 Snellen lines was achieved in 27 eyes (90%), while the remaining 3 eyes (10%) maintained stable vision. No patient experienced a decrease in BCVA during the 2-year follow-up. Exploratory sex-stratified analysis showed no significant difference in 24-month BCVA between male (0.24 ± 0.15 LogMAR) and female (0.22 ± 0.13 LogMAR) patients (*p* = 0.61). IOP values were similarly comparable (*p* = 0.54). The distribution of visual acuity outcomes is shown in Figure 2. Boxplot analysis demonstrated a marked overall improvement in BCVA from baseline to all postoperative time points, with a progressive shift toward lower (better) LogMAR values. The interquartile range narrowed substantially after the first postoperative month, reflecting increased homogeneity of visual outcomes across the cohort. Variability was greatest preoperatively (IQR = 0.30 LogMAR) and reached its lowest value at 24 months (IQR = 0.12 LogMAR). No extreme outliers were observed at any visit, supporting the consistency and reproducibility of the needle-guided single-suture technique.

All IOLs were well-centered and stable throughout the postoperative period. Anterior segment OCT and slit-lamp evaluations revealed no cases of tilt or decentration >0.3 mm. The mean IOL tilt angle, measured in 10 randomly selected eyes using anterior segment OCT, was 2.1 ± 0.7°, within normal limits.

The mean preoperative IOP was 15.2 ± 3.4 mmHg, increasing slightly to 17.1 ± 3.9 mmHg on postoperative day 10, returning to baseline levels (15.6 ± 2.8 mmHg) by 1 month (*p* = 0.12, not significant). No patient developed sustained ocular hypertension or hypotony (<6 mmHg).

Postoperative findings were mild and self-limited: conjunctival hyperemia: 21 eyes (70%), resolving within 2–3 weeks with topical therapy, mild corneal edema: 3 eyes (10%), resolving within 7–10 days, transient anterior chamber inflammation (grade 1): 5 eyes (16.7%), responding to corticosteroids. No cases of endophthalmitis, vitreous hemorrhage, cystoid macular edema, or retinal detachment occurred during follow-up. Suture exposure or erosion was not observed in any case.

The mean surgical time was 29.4 ± 4.8 min, significantly shorter than traditional double-suture scleral fixation techniques reported in the literature (~40–50 min) [20,21,22,23]. After the first 5 cases, operative duration stabilized around 27 min, indicating a short learning curve.

## 4. Discussion

This retrospective case series demonstrates that the needle-guided single-suture scleral fixation technique provides excellent IOL stability, visual rehabilitation, and a favorable safety profile over 24 months. The improvement from a mean preoperative BCVA of 0.89 LogMAR to 0.23 LogMAR at two years represents a mean visual gain of 0.66 LogMAR, comparable to outcomes reported in studies of Yamane’s flanged intrascleral fixation (0.7 LogMAR improvement) [16] and glued IOL methods (0.6–0.8 LogMAR) [17]. The consistent postoperative stability observed underscores the optical quality achieved by accurate IOL centration.

All lenses remained centered, and the absence of tilt or decentration aligns with the low variability reported in recent Carlevale and Yamane series (tilt < 3° in 95% of cases) [18,24]. The single transscleral fixation point provides symmetrical support through equal tension distribution, avoiding torsional stress typical of double-suture or haptic-externalization techniques [20]. The measured IOL tilt of 2.1 ± 0.7° confirms the method’s geometric precision, supported by the use of a 24-gauge needle to guide suture trajectory. The scleral tunnels’ tangential orientation prevents inadvertent vitreous entry, further minimizing risk of tractional complications.

The absence of serious postoperative complications—particularly retinal detachment, endophthalmitis, or persistent inflammation—highlights the atraumatic nature of this approach. Traditional sutured fixations report retinal detachment rates up to 2–5% and suture erosion in 6–10% of cases [25,26]. None of these occurred in the present cohort, likely due to the intralamellar tunnel technique, which fully buries the knot under conjunctiva. The transient postoperative hyperemia (70%) and anterior chamber inflammation (16.7%) observed were mild and expected, consistent with early healing responses seen in prior series [21,24].

A defining advantage of this method lies in its short learning curve. After fewer than five cases, operative time plateaued at ~27 min, and intraoperative difficulties decreased notably. In comparison, Yamane’s double-needle approach often requires more than 20 cases to achieve proficiency [16,20]. The needle-guided single-suture fixation is also instrument-economical. It requires only standard microsurgical tools and sutures, eliminating the need for vitrectomy ports, glue, or custom IOLs. This simplicity makes the procedure particularly suitable for surgeons in training or centers without vitreoretinal facilities. Compared to published results, our outcomes show similar or superior IOL centration and comparable visual gains with fewer procedural requirements (Table 2) [16,17,18,19,24,27].

Biomechanically, the single-suture technique redistributes tension along the scleral tunnel, forming a stable pivot that secures both haptics evenly. This avoids asymmetric force vectors, one of the main contributors to postoperative IOL tilt in two-point fixation systems [23]. The use of polypropylene 10-0 sutures has historically raised concerns of late degradation beyond 10 years [26]; however, encapsulation of the suture within the scleral wall substantially reduces exposure to UV and enzymatic degradation. Alternative materials, such as expanded polytetrafluoroethylene (ePTFE, Gore-Tex), offer improved long-term tensile strength [28], and future adaptations of this technique could incorporate such sutures for enhanced durability.

At two years, none of the 30 eyes exhibited IOL displacement or suture failure. This finding compares favorably with 3–5% dislocation rates reported for sutureless and glued methods beyond 18 months [18,21]. The tunnel-based knot burial and minimal conjunctival manipulation likely contribute to reduced postoperative fibrosis and stable fixation.

A key strength of this technique lies not only in its clinical outcomes but also in its educational and economic feasibility. In many institutions, especially within public health systems or residency programs, the availability of vitreoretinal-trained staff or advanced instrumentation may be limited. The needle-guided single-suture fixation provides an entry-level approach for anterior segment surgeons, offering reproducible results with minimal risk of major complications. The technique’s short learning curve and use of standard ophthalmic tools make it an attractive addition to surgical curricula.

Economically, the cost reduction compared to sutureless or glue-assisted fixation is substantial. The elimination of fibrin glue, customized haptic lenses, or specialized cartridges reduces operative expenditure by up to 40%, according to cost analyses of similar procedures [23,27]. In resource-constrained regions, this represents a critical advantage that can facilitate broader access to secondary IOL implantation. From a technological standpoint, the field of IOL fixation is rapidly progressing toward integration with digital guidance and robotic microsurgery. Three-dimensional visualization systems and intraoperative OCT now enable real-time assessment of tunnel positioning and IOL centration. Future iterations of the needle-guided approach could incorporate these tools to further improve accuracy and reproducibility [29]. Furthermore, experimental materials such as ePTFE (Gore-Tex) and ultra-high-molecular-weight polyethylene sutures are under investigation for their resistance to degradation and elongation. Combining these materials with the single-suture concept may extend the longevity of fixation and reduce late postoperative complications. The simplicity of the current method makes it a promising platform for such innovations, providing a foundation for future refinements in minimally invasive IOL fixation. The clinical versatility of the needle-guided single-suture technique extends beyond standard aphakia management. It can be successfully applied in complex ocular scenarios, such as post-vitrectomy eyes, traumatic aniridia, or cases involving keratoprosthesis-associated IOL instability. The ability to perform the entire procedure through a small corneal incision without requiring vitreoretinal instrumentation reduces ocular stress and shortens rehabilitation. Furthermore, by preserving conjunctival integrity and minimizing scleral disruption, the method maintains a favorable ocular surface environment, which may facilitate future glaucoma filtration or keratoplasty procedures if needed. The observed stability at 24 months supports its application in multimorbid elderly patients, where safety and efficiency are paramount. Integrating this approach into broader anterior segment surgical algorithms could standardize secondary IOL implantation protocols, bridging the gap between high-technology sutureless systems and traditional manual techniques, while maintaining excellent functional and anatomical outcomes.

The main limitations of this study are its retrospective design, limited sample size, and lack of a comparative control group. Furthermore, postoperative imaging was limited to slit-lamp and anterior segment OCT rather than Scheimpflug-based tilt quantification in all cases. Scheimpflug-based tilt measurements were not uniformly performed, restricting our ability to quantify IOL tilt with the same precision reported in comparative studies. These methodological constraints limit definitive biomechanical validation of the technique when compared with standard approaches such as Yamane or Carlevale fixation. This study did not evaluate also cellular or biochemical parameters such as posterior capsular opacification, residual lens epithelial cell proliferation, or long-term tissue–suture interactions. These aspects remain important to fully understand long-term biocompatibility of the technique and should be addressed in future prospective studies. Nonetheless, the consistent results across all patients and the 24-month follow-up period provide strong preliminary evidence for the technique’s reproducibility. Future prospective, randomized multicentric studies comparing this single-suture needle-guided method against Yamane and Carlevale techniques would be valuable to validate its long-term biomechanical integrity and cost-effectiveness. Future studies should include randomized multicenter trials directly comparing this method with established techniques such as Yamane and Carlevale fixation. Additionally, testing alternative sutures such as ePTFE (Gore-Tex) may offer improved long-term durability and reduced degradation compared with polypropylene.

## 5. Conclusions

The present study demonstrates that the needle-guided single-suture scleral fixation technique is a safe, efficient, and reproducible approach for secondary intraocular lens implantation in eyes lacking adequate capsular support. With a mean BCVA improvement of 0.66 LogMAR, zero cases of IOL tilt or dislocation, and no major intraoperative or postoperative complications over two years, this method offers outcomes comparable to or exceeding those of established techniques such as Yamane’s flanged or Carlevale’s intrascleral fixation.

The mean surgical time of 29 min and minimal learning curve further underscore its practicality for both experienced and junior anterior segment surgeons. The reliance on standard microsurgical instruments and materials makes it cost-effective and suitable for widespread clinical adoption, including resource-limited settings. By simplifying scleral fixation while preserving anatomical precision, the needle-guided single-suture method represents a valuable addition to the evolving landscape of intraocular lens implantation techniques. Further long-term and comparative studies will help consolidate its role in modern ophthalmic surgery.

Beyond its immediate clinical impact, the needle-guided single-suture scleral fixation technique represents an evolving paradigm that balances surgical precision, accessibility, and educational value. As technology and materials advance, this method may serve as a cornerstone for next-generation scleral fixation models integrating digital guidance and long-lasting suture innovations.

## Figures and Tables

**Table 1:** Baseline demographic and clinical characteristics of the study population.

Characteristic	Value
Number of patients (eyes)	30 (30)
Age (years), mean ± SD (range)	66.4 ± 9.7 (48–82)
Sex distribution	18 males (60.0%), 12 females (40.0%)
Primary indication for surgery	
• Dislocated intraocular lens (IOL)	12 eyes (40.0%)
• Post-traumatic aphakia	9 eyes (30.0%)
• Zonular dialysis after complicated cataract surgery	7 eyes (23.3%)
• Congenital ectopia lentis	2 eyes (6.7%)
Baseline BCVA (LogMAR)	0.89 ± 0.32
Baseline IOP (mmHg)	15.2 ± 3.4
Follow-up duration (months)	24.3 ± 2.1 (range 22–27)

**Table 2:** Comparison with other techniques.

Technique	Mean BCVA Gain (LogMAR)	IOL Tilt Rate	Key Advantages	Limitations
Traditional double-suture	0.5–0.6	10–15%	Widely available	Suture erosion, long surgery
Yamane double-needle	0.7	10%	Sutureless, stable	Steep learning curve
Glued IOL (Agarwal)	0.6–0.8	8–10%	Strong fixation	Biological glue handling
Carlevale lens	0.7	<5%	Preloaded lens design	Expensive, specific IOL
Needle-guided single-suture (current)	0.66	0% (≤2° tilt)	Simple, safe, fast	Requires precise needle alignment

## Data Availability

The original contributions presented in this study are included in the article. Further inquiries can be directed to the corresponding author.

## Abbreviations

The following abbreviations are used in this manuscript:

IOL	Intraocular Lens
